# Docking to a Basic Helix Promotes Specific Phosphorylation by G1-Cdk1

**DOI:** 10.3390/ijms22179514

**Published:** 2021-09-01

**Authors:** Ilona Faustova, Kaidi Möll, Ervin Valk, Mart Loog, Mihkel Örd

**Affiliations:** Institute of Technology, Faculty of Science and Technology, University of Tartu, 50411 Tartu, Estonia; ilona.faustova@ut.ee (I.F.); kaidi.moll@ut.ee (K.M.); ervin.valk@ut.ee (E.V.)

**Keywords:** cyclin specificity, cyclin-dependent kinase, SLiM, kinase specificity, phosphorylation

## Abstract

Cyclins are the activators of cyclin-dependent kinase (CDK) complex, but they also act as docking scaffolds for different short linear motifs (SLiMs) in CDK substrates and inhibitors. According to the unified model of CDK function, the cell cycle is coordinated by CDK both via general CDK activity thresholds and cyclin-specific substrate docking. Recently, it was found that the G1-cyclins of *S. cerevisiae* have a specific function in promoting polarization and growth of the buds, making the G1 cyclins essential for cell survival. Thus, while a uniform CDK specificity of a single cyclin can be sufficient to drive the cell cycle in some cells, such as in fission yeast, cyclin specificity can be essential in other organisms. However, the known G1-CDK specific LP docking motif, was not responsible for this essential function, indicating that G1-CDKs use yet other unknown docking mechanisms. Here we report a discovery of a G1 cyclin-specific (Cln1,2) lysine-arginine-rich helical docking motif (the K/R motif) in G1-CDK targets involved in the mating pathway (Ste7), transcription (Xbp1), bud morphogenesis (Bud2) and spindle pole body (Spc29, Spc42, Spc110, Sli15) function of *S. cerevisiae*. We also show that the docking efficiency of K/R motif can be regulated by basophilic kinases such as protein kinase A. Our results further widen the list of cyclin specificity mechanisms and may explain the recently demonstrated unique essential function of G1 cyclins in budding yeast.

## 1. Introduction

Cyclin-dependent kinases (CDK), hierarchically the most central regulatory hubs of cell division, are activated by different cyclins at different cell cycle stages. CDK complexes organize and control all essential processes of cell division including chromosome duplication, cytoskeletal reorganizations, mitotic spindle dynamics, sister chromatic separation, cytokinesis and others [1].

Phosphorylation of hundreds of CDK targets, most of them at multiple S/TP consensus motifs, is part of an enormous coordination task, whose flawless order is the key for cell survival, fitness and errorless progeny [2]. Recent studies have developed a unified model of CDK function that combines the two initial models: the quantitative or the threshold model, and the cyclin specificity model. The quantitative model states that cell cycle events are ordered by accumulating or declining cyclin levels at different CDK activity thresholds [3,4,5]. Early phosphorylation events take place at lower cyclin levels and lower CDK activity thresholds. The cyclin specificity model also takes into account the cyclin-specific substrate recognition at different cell cycle stages. The principle of the quantitative model was elegantly demonstrated in a fission yeast strain that was able to drive the cell cycle using just a single mitotic cyclin [6]. The cyclin specificity, on the other hand, has been demonstrated for all major cyclins in budding yeast and also for several CDK complexes in mammalian cells [7,8,9,10].

One of the key elements of the unified model is that the active site-specificity of CDK, measured using a consensus phosphorylation site peptide, is gradually increasing in the order of the appearance of the cyclins in the cell cycle [11]. The K_M_ values are decreasing and the k_cat_/K_M_ values increasing, making the early CDK complexes relatively weak kinases. This is both the case in budding yeast and human CDK complexes [11,12]. Such gradual change likely provides the first coarse mechanism for preventing premature triggering of the mitotic events by early CDKs. Secondly, in the case of several early CDK targets, the poor intrinsic activity of early complexes is compensated by phosphorylation mechanisms involving cyclin-specific substrate docking. For example, in budding yeast, the G1 cyclins Cln1,2 have been shown to dock a hydrophobic leucine-proline rich sequence designated as LP motif [11,13,14], the S-phase cyclin Clb5 uses at least two docking motifs: the classical R/KxL motif [15,16,17,18], and recently discovered NLxxxL motif [19], and the G2 cyclin Clb3 binds a motif PxxPxF [20]. Finally, despite the high intrinsic activity, the specificity of mitotic targets for M-CDK (Clb2,1-Cdk1) complex can be considerably improved using short linear motifs with minimal consensus LxF [21] In addition to the changes in CDK activity and specificity over the cell cycle, the counteracting phosphatases also have varying activity towards different CDK targets and this contributes to the timing of different phosphorylation and dephosphorylation switches [22,23,24,25].

Another crucial element that controls the phosphorylation thresholds of many targets is the phospho-adaptor subunit of the CDK complex, the Cks1 [26,27,28]. The phospho-pocket of Cks1 docks the previously phosphorylated threonines and thereby facilitates the phosphorylation of secondary C-terminally located sites [27]. Most of the CDK targets have multiple phosphorylation sites in intrinsically disordered regions [29], and thus, these networks of sites together with the cyclin docking sites and Cks1 priming phosphorylation sites form a “linear barcode” that is read and processed by the cyclin-Cdk1-Cks1 complex that acts as a structured catalytic scaffold [28]. Different parameters such as optimal distances between docking sites and phosphorylation sites, distribution of serines and threonines in CDK consensus sites together with other specificity elements form a multisite phosphorylation code that controls the net rate of phosphorylation of the network. This net rate defines the general CDK threshold for a target, and thereby also the timing of the CDK-driven cell cycle switch. By analogy, the multisite phosphorylation networks in a CDK target can be compared to resistors in electronics: the better the parameter set, or lower the resistance, the more efficiently the CDK complex can read the multisite phosphorylation code, leading to a faster net rate of phosphorylation. When the parameter set is not optimal, the resistance is higher and the CDK threshold is higher, designating the CDK switch to take place later in the cell cycle.

A recent elegant study has nicely demonstrated the essence of the unified model of CDK function by discovering that budding yeast requires the specificity of G1 cyclins to perform a specific function in polarization and bud growth, while the S and M phases can be driven by a single mitotic cyclin [30]. Thus, the G1 cyclins’ essentiality for cell survival requires cyclin specificity not present in mitotic cyclin Clb2. However, interestingly, this essential function for budding and cell proliferation was not dependent on the known leucine-proline rich (LP-motif) motif as the Cln2 version with mutated LP motif binding pocket did not show any deviation compared to the wild-type Cln2 [30,31]. Thus, the authors concluded that the features of Cln2 that are responsible for the phosphorylation specificity behind this essential function are yet to be discovered as they must lie outside its LP motif docking site.

In the present study, we demonstrate a specific substrate docking mechanism for Cln1,2 complex that is different from the LP substrate docking mechanism. We discovered that a G1 cyclin-specific (Cln1,2) lysine-arginine-rich helical docking motif (the K/R motif) in G1-CDK substrates potentiates phosphorylation of G1-specific targets involved in the mating pathway, G1/S transcription, bud morphogenesis and spindle pole body function of *Saccharomyces cerevisiae*. Using several biochemical and in vivo approaches, we demonstrate the role of K/R docking in bud morphogenesis, spindle pole body regulation and dynamic localization cycle of Sli15, a subunit of the Ipl1/Aurora B kinase complex. We also demonstrate a possibility to modulate potency of the helical K/R docking module by protein kinase A catalyzed phosphorylation of adjacent sites, adding yet another input and regulatory layer that the cell can use to tune the CDK thresholds in response to different stimuli.

## 2. Results

### 2.1. A Set of G1 Cyclin Specific Cdk1 Targets Are Independent of the LP Docking

**Figure 1:**
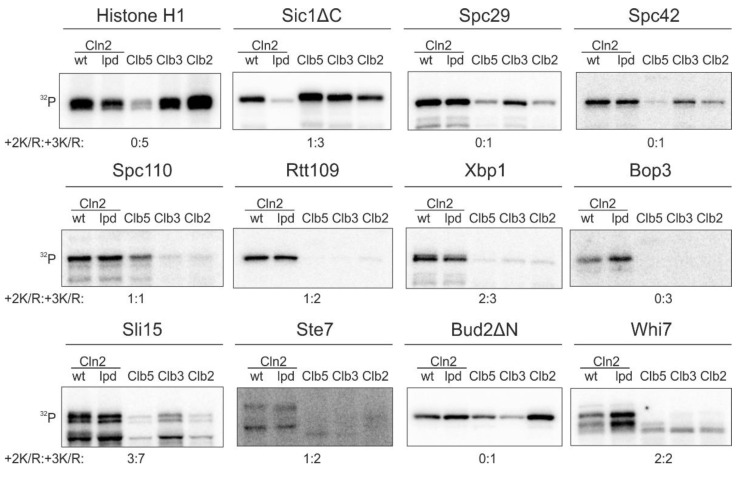
A substrate docking interaction different from the known LP interaction, governs the phosphorylation specificity of a group of key targets of G1-Cdk1. ^32^P-autoradiographs showing phosphorylation of Cdk1 targets by the four major cyclin-Cdk1 complexes and the LP docking deficient Cln2(lpd)-Cdk1. +2K/R:+3K/R shows the number Cdk1 consensus phosphorylation sites with basic residue in +2 position and +3 position. The experiments were performed twice, a representative example is shown.

While the Cln1,2-specific LP docking motif is important for phosphorylation of key G1 targets [11,13,14], our quantitative phosphorylation studies indicated that a fraction of known G1-specific Cdk1 targets does not use this docking interaction (Figure 1). We measured the cyclin specificity of these G1-Cdk1 targets using in vitro kinase assays with the major cyclin-Cdk1 complexes. To gain an insight into the LP docking dependence, we used a Cdk1 complex with Cln2-lpd mutant that does not interact with LP motifs [31]. The Cln2(lpd)-Cdk1 complex phosphorylated Sic1ΔC, a substrate with an LP motif [11,13], 5 times slower than the wild-type complex (Figure 1). Even though LP docking mediates phosphorylation of a large variety of G1-Cdk1 targets, our screen revealed 10 substrates that were specifically phosphorylated by G1-Cdk1, but whose phosphorylation was not affected by the loss of LP docking (Figure 1). These included spindle pole body components Spc29, Spc42 and Spc110, transcriptional regulators Xbp1, Rtt109 and Whi7, kinetochore-microtubule regulator Sli15, bud morphogenesis protein Bud2 and others.

In addition to LP docking, G1 cyclin specificity has been shown to arise from changes in CDK active site specificity. In comparison to minimal consensus phosphorylation sites (S/T-P), phosphorylation sites with a basic residue in +3 position (S/T-P-x-K/R) are generally preferentially targeted by Cdk1 [32,33]. While a basic residue in +2 position does not significantly affect phosphorylation by Clb-Cdk1 complexes, it greatly enhances phosphorylation by Cln1,2-Cdk1 [11]. Many of the discovered Cln2-specific targets (Figure 1), however, do not contain such phosphorylation sites, indicating that there is yet an undefined specificity mechanism that directs efficient phosphorylation of G1-Cdk1 targets.

### 2.2. A Basic Substrate Docking Motif Directs Specific Phosphorylation of Early Cell Cycle Targets by G1-Cdk1

**Figure 2:**
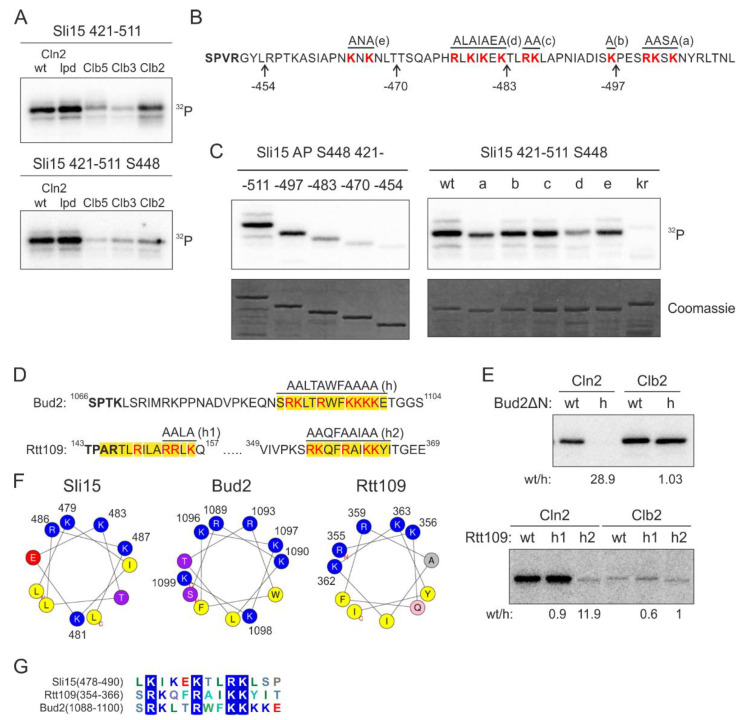
Clusters of basic residues promote phosphorylation by G1-Cdk1. (**A**) ^32^P-autoradiographs of phosphorylation reactions of Sli15 fragment 421–511 with all phosphorylation sites (upper panel) or with just S448 as the only Cdk1 consensus site by different cyclin-Cdk1 complexes in vitro. (**B**) Sequence of Sli15 positions 448–511 showing the truncations and mutations used in panel C. (**C**) Phospho-images showing phosphorylation of the Sli15 mutants by Cln2-Cdk1. The mutants are described in ‘B’. In the kr mutant, all highlighted K/R residues are mutated to N/Q residues, respectively. (**D**) Sequences of the predicted α-helical K/R docking motifs in Bud2 and Rtt109. (**E**) ^32^P-autoradiographs showing the phosphorylation of Bud2 and Rtt109 proteins by Cln2- and Clb2-Cdk1. wt/h shows the relative phosphorylation rate of the wild-type substrate protein compared to the K/R docking site mutant. (**F**) Helical wheel representations of the K/R docking motifs in Sli15, Bud2 and Rtt109 show the clustering of basic residues on one side of the α-helix. (**G**) Multiple sequence alignment of K/R motifs in Sli15, Bud2 and Rtt109.

We set out to study this yet uncharacterized docking mechanism using Sli15, the subunit of yeast Aurora B kinase Ipl1, as a model substrate. We found that the Cln2-specificity was retained in a smaller Sli15 fragment containing positions 421–511 and 5 full consensus Cdk1 phosphorylation sites. To further simplify the model substrate, we introduced alanine mutations into the four Cdk1 consensus sites except for S448, which was efficiently phosphorylated in this protein fragment (Figure 2A). Interestingly, the majority of Cln2-Cdk1 activity towards Sli15(421–511) was directed to S448, while S427 is phosphorylated at a 10 times lower rate (Figure S1A). In the case of Clb5- and Clb2-Cdk1, the two sites are targeted with similar efficiency, indicating that the docking interaction directs Cln2-Cdk1 to preferentially phosphorylate S448.

Next, to map the interaction, we created a series of C-terminally truncated Sli15 fragments (Figure 2B) and analyzed their phosphorylation by Cln2-Cdk1. Interestingly, the experiments revealed a gradual decrease in the phosphorylation rate of these fragments by Cln2-Cdk1 (Figure 2C). This indicates that the Cln2-specificity, in this case, does not arise from a single short linear motif (SLiM).

As all of the truncated segments contained clusters of basic residues, we hypothesized that they might be involved in docking (Figure 2B). Next, we created 5 different mutants of the positively charged residues in the segments and found that 3 different mutations caused a considerable decrease in phosphorylation rates (Figure 2C). Interestingly, this region is predicted to contain an α-helix in positions 478–488 and the K/R residues spaced 2–3 amino acids apart fall on one side of the α-helix. Further, combined mutation of all these basic residues resulted in over 30-fold decrease in Cln2-specificity (Figure S1B). This indicates that the clusters of basic residues, either as a disordered segment or an amphipathic α-helix where the basic residues are on one side of the helix, might function as a docking module.

The interaction was specific for Cln2 compared to Clb cyclins, as mutation of the basic residues only slightly affected Sli15(421–511) phosphorylation by Clb5- and Clb2-Cdk1 (Figure S1B). To test the impact of the docking on the phosphorylation, we measured the Michaelis-Menten kinetics of wild-type Sli15(421–511) and the kr mutant, where the K/R residues highlighted in Figure 2B are mutated to N/Q. Mutation of the basic residues in Sli15 resulted in a profound increase in K_M_, confirming the loss of kinase-substrate docking (Figure S1C). Even though the predicted helix was the central most important element of the docking motif, as mutation of these residues led to the greatest decrease in phosphorylation rate (Figure 2C), the additional clustered K/R residues nearby contributed to substrate phosphorylation as well. The number of basic amino acids in the disordered stretch is likely a flexible evolutionary route to set a range of different thresholds as we observed about 15 times difference in k_cat_/K_M_ values within the list of K/R substrates tested (Figure 1).

The other targets studied in detail included a histone acetyltransferase Rtt109, and Bud2, a protein with a role in bud site selection [34,35]. Analogously with Sli15, in the case of Bud2, the mutation of a candidate helix caused a considerable reduction of phosphorylation rates in the case of Cln2, but not with Clb2 complex (Figure 2D,E). In Rtt109, two candidate motifs were mutated, with only one of the two exhibiting loss of phosphorylation, while the other, being possibly too close to the phosphorylation site, showed no effect (Figure 2D,E and Figure S1D). Analogously, in our previous studies on cyclin docking motifs, we have found that a certain minimal distance between the docking site and the phosphorylation site is a cutoff for Cdk1-dependent phosphorylation [27]. In all three model substrates, and in several other substrates from the phosphorylation screen, the docking motif was predicted to form an α-helix with basic residues clustering on one side of the helix, presumably creating a positively charged docking area (Figure 2F). As the characterized motifs in Sli15, Bud2 and Rtt109 share little similarities other than the K/R residues (Figure 2G) and mutation of the K/R residues resulted in a profound decrease in phosphorylation rate by Cln2-Cdk1, we named the novel docking motif as K/R motif.

### 2.3. Mutation of a Docking Site on Cyclin Affects Selectively the K/R Docking Mechanism but Not the LP Docking Mechanism

**Figure 3:**
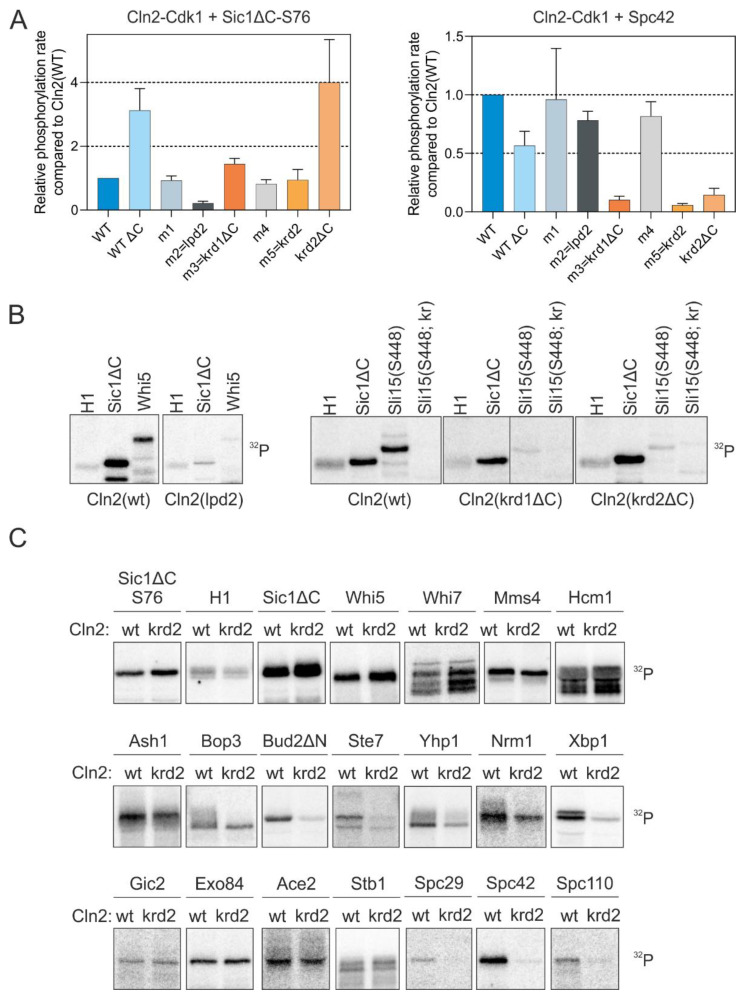
Cln2 mutants deficient in either LP or K/R docking. (**A**) The docking specificity of mutant Cln2-Cdk1 complexes was analyzed in vitro by measuring phosphorylation of LP-dependent substrate Sic1-S76 and K/R-dependent Spc42. The mutations in each Cln2 mutant and their position on the Cln2 surface are shown in Figure S2A,B. Cln2-ΔC indicates deletion of the C-terminal intrinsically disordered domain of Cln2 (positions 372–545). (**B**) ^32^P-autoradiographs showing the phosphorylation of histone H1, Sic1ΔC, Whi5, Sli15(421–511 S448) and Sli15(421–511 S448; kr), where the basic residues involved in K/R docking (highlighted in Figure 2B) are mutated to Q and N, by different Cln2-Cdk1 mutant complexes. (**C**) The effect of K/R docking on phosphorylation of various G1-Cdk1 targets was studied in vitro using Cln2(krd2) mutant. Representative ^32^P-autoradiographs are shown.

Using systematic mutagenesis of combinations of residues on the Cln2 surface, we were able to map the regions on Cln2 responsible for docking the K/R motif. We targeted regions where conserved polar residues are exposed on the Cln2 surface, introduced alanine mutations into these residues and purified the mutant Cln2-Cdk1 complexes (Figure S2). To test the docking specificity of different Cln2 mutants, we measured the phosphorylation rate of Sic1, a known LP-dependent substrate [11,13], and Spc42, a Cln2-specific substrate whose phosphorylation is not affected by mutation of the LP pocket (Figure 1). Deletion of the intrinsically disordered C terminus of Cln2 mildly affected the docking specificity of the Cdk1 complex, as Cln2ΔC had slightly higher activity towards Sic1 and slightly decreased activity towards Spc42, compared to the wild-type complex (Figure 3A). Cdk1 in complex with one of the Cln2 mutants (Cln2-m2 (^63^DQQPEMN^69^, where the underlined residues are mutated to alanine)) was unable to efficiently phosphorylate Sic1ΔC and Whi5, but not Spc42, indicating the absence of LP docking (Figure 3A,B and Figure S2). Cln2-m2 contained mutations in the vicinity of the known LP docking interface [31], suggesting that LP motifs bind to a slightly wider area on the cyclin. Specific loss of Spc42 phosphorylation rate was observed with two Cln2 mutants, Cln2-m3(krd1ΔC (^209^NCLMQYE^215^ with deletion of the disordered C terminus 373–545 to improve protein purification)) and Cln2-m5(krd2 (^334^PNSLME^339^, where the underlined residues are mutated to alanine)) (Figure 3A and Figure S2). Mutation of either of these sites caused a loss of phosphorylation similar to the case of the K/R mutation in Sli15 (Figure 3B). These mutations were entirely specific for the docking mechanism as they did not affect the overall activity of the Cdk1 complex as judged from the phosphorylation of reference substrate Histone H1 (Figure 3B). However, the mutations in krd1 and krd2 do not point to a specific well-defined pocket on Cln2 that binds the K/R motif, as the mutations that affect K/R docking are distributed on a larger area. Thus, further studies are required to precisely map the docking interaction from the cyclin side.

Next, we tested a larger set of targets with one of the docking mutants (Cln2-krd2). While the targets using solely the LP motif for cyclin docking (Sic1ΔC and Whi5) and a number of Cln2-Cdk1 targets that do not have validated LP motifs (Whi7, Mms4, Hcm1) were not affected by the Cln2 mutation, several targets carrying the K/R-motif, including several spindle pole body (SPB) components, Bud2 and some other known G1-Cdk1 targets showed a strong effect (Figure 3C). Interestingly, phosphorylation of Whi7 by Cln2-Cdk1 was not affected by the loss of either LP or K/R docking (Figure 3C), indicating that the G1 cyclins could contain yet another substrate interaction, potentially mediated by substrate docking to Cdk1 [36].

### 2.4. Potential Role of K/R Docking in Bud Morphogenesis and Spindle Pole Body Regulation

Next, we set out to describe the importance of the docking interactions in the G1 cyclin function. We first confirmed that the three novel Cln2 mutants are all expressed when replaced into *CLN2* endogenous locus, with Cln2(krd1) accumulating to a higher level than the wild-type (Figure S3A). To confirm the functionality of these mutants in vivo, we studied the phosphorylation of Whi7 in the cell cycle, as phosphorylation of Whi7 is not affected by LP or K/R docking (Figure 1 and Figure 3C). The peak of Cln2 expression was 30–45 min after release from pheromone-induced G1 arrest and this is accompanied by accumulation of phosphorylated Whi7 in the strains that express Cln2 (Figure S3B). The identified Cln2-lpd2 and krd mutants promoted Whi7 phosphorylation similarly to the wild-type Cln2 (Figure S3B), verifying the activity of these Cln2-Cdk1 complexes. Cyclins deficient in either LP or K/R docking were capable of driving cell cycle progression as the only G1 cyclin (Figure S3C), indicating that these docking interactions separately are not essential in promoting cell cycle entry.

**Figure 4:**
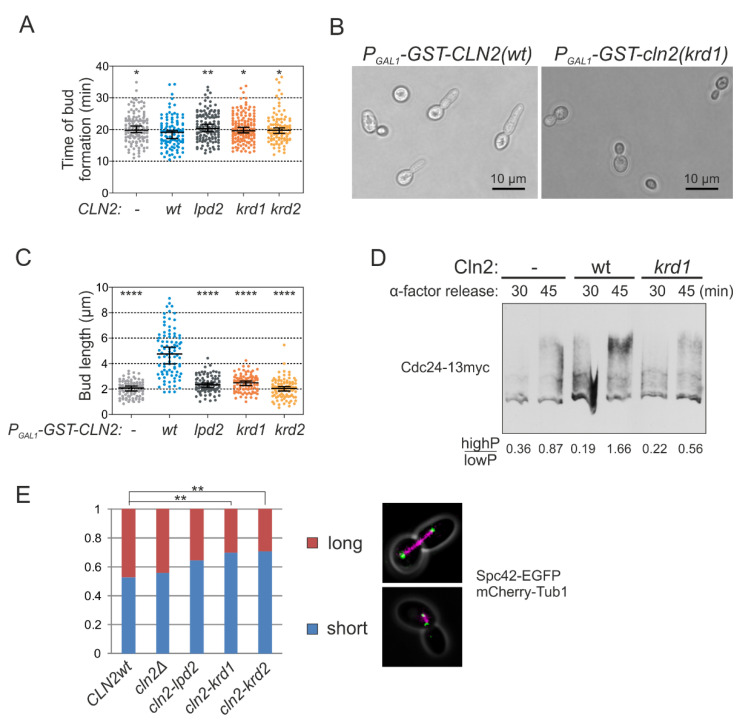
K/R docking mediates Cln2 function in bud morphogenesis and spindle pole body dynamics. (**A**) The time from Start, measured by the nuclear export of Whi5-mCherry, to budding was studied in time-lapse microscopy in single cells of *cln1*Δ strain expressing the indicated *CLN2* variant from *CLN2* promoter. The error bars show 95% confidence intervals of the median. * indicates *p*-value < 0.05 and ** *p*-value < 0.01 in pairwise comparisons of the indicated condition with *CLN2(wt)* by Mann-Whitney U-test. (**B**) Microscopy images showing the effect of GST-Cln2 overexpression on bud morphology. (**C**) Plot showing the capability of different Cln2 variants to promote elongated bud growth. The bud length was measured 2 h after *P_GAL1_-CLN2* induction. **** indicates *p*-value < 0.0001 for pairwise comparisons with *CLN2(wt)* using Mann-Whitney U-test. (**D**) Multisite phosphorylation of Cdc24-13myc was studied by Phos-tag Western blotting of *cln1*Δ strains expressing the indicated *CLN2* variant from *CLN2* promoter. The cultures were synchronized in G1 using α-factor and were released to the cell cycle. Cdc24 phosphorylation was analyzed 30 and 45 min after releasing from α-factor arrest, at the peak of Cln2 expression. (**E**) The fraction of cells with short or long spindles was studied with fluorescence microscopy in *cln1*Δ strains expressing different Cln2 mutants, Spc42-EGFP and Tub1-mCherry. ** denotes *p* < 0.01 for comparison with *CLN2wt* by χ^2^-test.

As the G1 cyclins drive bud formation [37], we measured the time of bud formation from Start and observed a slight delay in the case of both lpd and krd Cln2 mutants (Figure 4A). As Cln2-Cdk1 promotes bud elongation, in contrast to Clb-Cdk1, overexpression of Cln2 causes elongated bud morphology [31,37]. Overexpression of the docking interface mutants did not cause the elongated bud phenotype characteristic of wild-type *CLN2* overexpression indicating that both LP and K/R docking mechanisms have a role in phosphorylating substrates involved in polarized growth (Figure 4B,C). A key Cdk1 substrate in triggering bud formation is Cdc24 [2,38]. To test whether K/R docking mediates phosphorylation of Cdc24, we performed Western blot from synchronized cultures using Phos-tag SDS-PAGE to separate protein forms based on phosphorylation. These experiments showed that Cdc24 is phosphorylated in the early cell cycle, but the phosphorylation is considerably decreased in cells expressing Cln2 K/R docking mutant (Cln2(krd1)) at endogenous level in *cln1*Δ strain (Figure 4D). Thus, both LP and K/R docking interactions direct G1-Cdk1 to phosphorylate substrates to regulate bud formation.

For another example, we addressed the role of K/R-mediated interactions in SPB function as a number of K/R docking targets were SPB proteins (Figure 1 and Figure 3C). Cdk1-dependent phosphorylation of these SPB components has been suggested to control SPB duplication, separation and spindle formation [39,40,41]. For example, it has been shown that the cells carrying the version of Spc42 with mutated CDK sites have considerable delay or defects in spindle formation and elongation, which is caused by defects in SPB duplication [39]. We performed a similar analysis in large budded cells with *CLN2* gene replaced with the docking mutant and found a very similar shift in the distribution of short and long spindles as was observed in the referred study using Spc42 version with all CDK sites mutated to alanines (Figure 4E).Therefore, G1-Cdk1 could regulate the SPBs via K/R docking either directly through phosphorylation of SPB proteins or indirectly by affecting the activation of Anaphase Promoting Complex as a late consequence of earlier defects at G1/S.

### 2.5. The Spindle Localization of Sli15 of the Ipl1p/Aurora B Complex Is Phospho-Regulated via K/R Docking

**Figure 5:**
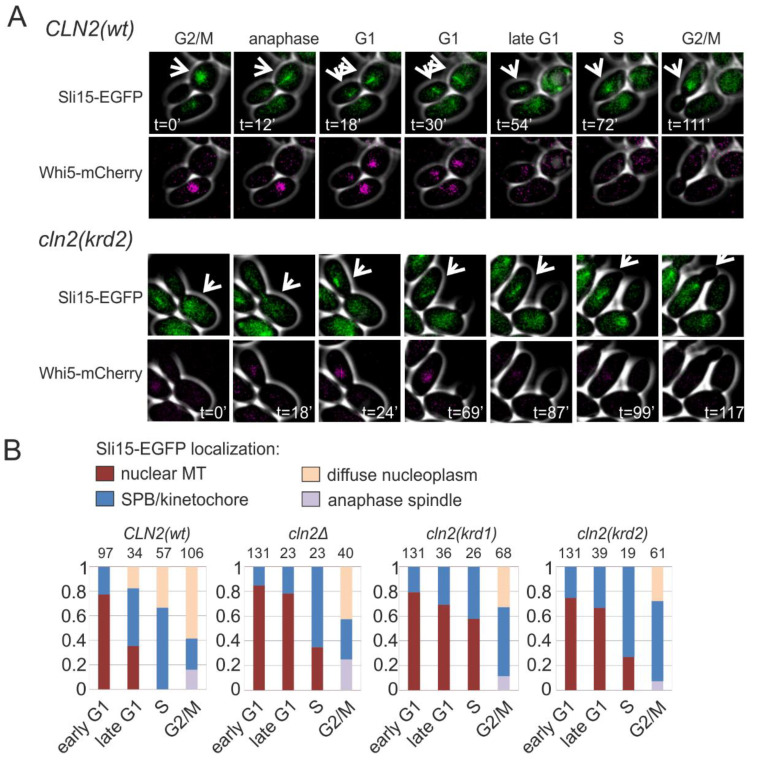
K/R docking regulates Sli15 localization dynamics. (**A**) Images showing the subcellular localization of Sli15-GFP during the cell cycle in cells expressing Cln2(wt) or Cln2(krd2). The nuclear localization of Whi5-mCherry was used as the reference for cell cycle progression. (**B**) Plots showing the fraction of cells with the indicated Sli15-GFP localization. Early G1 cells were unbudded cells with nuclear Whi5-mCherry, whereas late G1 cells were unbudded but without nuclear Whi5-mCherry signal. Small budded cells were denoted as S phase and large budded cells were categorized as G2/M cells. The experiments were performed using *cln1*Δ *cln2*Δ strains expressing the indicated *CLN2* variant under *CLN2* promoter on a centromeric plasmid.

In anaphase, Sli15, a component of the Ipl1p/Aurora B complex and a chromosomal passenger protein, translocates from the kinetochores to the spindle. In G1 it stays at the broken spindles in the nucleus (Figure 5A). At G1/S transition, Sli15 moves to the clustered spindle pole bodies and kinetochores, appearing as clear dots when expressed as GFP fusion protein (Figure 5A). In the S phase and G2/M, the localization of Sli15 spreads over the nucleoplasm. This dynamic cycle is Cdk1-regulated and we asked if the K/R docking mechanism and Cln2 could play role in the G1/S transition of Sli15 from spindle fragments to SPB/kinetochores. We followed the localization of Sli15-GFP in dividing cells and found that transition from nuclear microtubules to SPB/kinetochore foci was strongly delayed in both K/R docking mutants, as shown by the diagrams for different cell cycle stages in Figure 5B.

To observe the phosphorylation dynamics of Sli15 as a K/R docking-dependent substrate in vivo, we followed the phosphorylation shifts of a Sli15 fragment after the release from G1 arrest. The phosphorylation shifts were considerably lower in the case of mutant *CLN2* (Figure S4). Lower levels of phosphorylation were also observed in case the K/R motif was mutated in the Sli15 fragment. These data suggest that G1-CDK via the K/R docking of Sli15 regulates its localization cycle and thus also plays a role in the cell cycle function and activity profile of the Ipl1p/Aurora B complex.

### 2.6. Modulation of K/R Docking by PKA Phosphorylation

**Figure 6:**
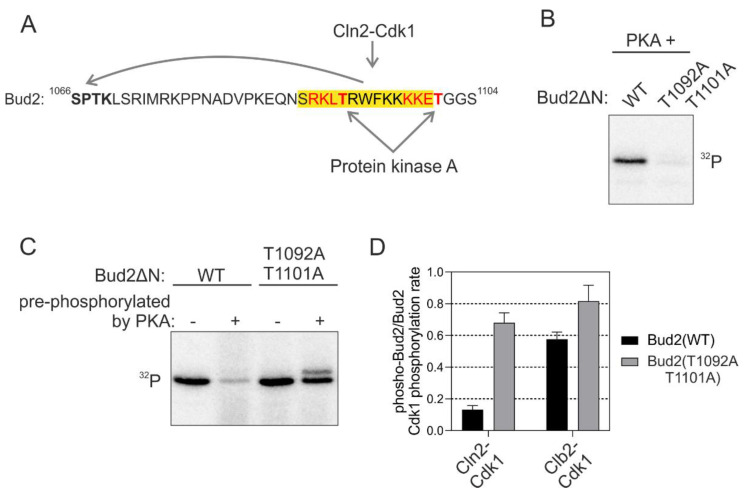
Phosphorylation adjacent to the K/R docking α-helix switches the docking off. (**A**) Scheme showing the sequence of Bud2 C terminus with two potential protein kinase A (PKA) phosphorylation sites in the K/R docking motif. (**B**) ^32^P-autoradiographs showing the phosphorylation of T1092 and T1101 by PKA. (**C**) The C terminus of Bud2 was first phosphorylated by PKA without ^32^P-ATP under conditions where Bud2 is expected to be fully phosphorylated at T1092 and T1101 by PKA, followed by removal of PKA and subsequent phosphorylation by Cln2-Cdk1 in the presence of ^32^P-ATP. “−“ indicates no pre-phosphorylation of Bud2ΔN, whereas “+” indicates that Bud2 ΔN has been pre-phosphorylated by PKA. An autoradiograph showing the inhibition of Bud2 phosphorylation by Cln2-Cdk1 by pre-phosphorylation of the substrate with PKA. (**D**) The effect of Bud2 pre-phosphorylation by PKA on subsequent phosphorylation by Cln2- and Clb2-Cdk1. The error bars show standard deviation.

As an additional intriguing element of the K/R docking mechanism, we observed that at the C-terminal side of several candidate K/R helixes, there were potential phosphorylation sites for basophilic kinases, with the consensus sequence matching the one of protein kinase A (PKA) (Figure 6A and Figure S5). It is generally known that phosphorylation, especially at the C-terminal end of α-helixes may cause the loss of helical structure [42]. We hypothesized that phosphorylation of these sites may cause the structure-unstructure switch that unwinds the helix and distorts the docking interaction.

In vitro kinase assays revealed that PKA phosphorylated the consensus sites in Bud2 K/R α-helix (Figure 6B). Next, we studied the docking-dependent phosphorylation of Bud2 by Cdk1 after the pre-phosphorylation with PKA. Severe loss of docking efficiency in wild-type substrate construct compared to the version with mutated PKA site, was observed (Figure 6C,D). No such effect was found in case Clb2-Cdk1 was used as the kinase, suggesting a strictly K/R and Cln-specific mechanism. Thus, the K/R docking mechanisms are not only enhancing the substrate interactions but can also function as a tunable interaction that controls a regulatory node integrating other kinase inputs. Such a conditional regulatory mechanism would be useful when a cell needs to adjust to conditions not favorable for cell cycle entry. The kinases signaling the information about such conditions would shift the thresholds up and delay the cell cycle entry until the conditions are favorable, or until the cell has activated an adaptation program. Indeed, a strong PKA signal causes a delay in G1 [43], and this could be one of the mechanisms of how it does it.

## 3. Discussion

While cyclins are known to be the activators of CDK complexes, a growing amount of evidence points that they also act as scaffold subunits for the CDK complex providing binding pockets to accommodate different substrate and inhibitor docking motifs. As the early cyclin complexes have lower intrinsic kinase activity [11,12,18], these substrate docking interactions have important roles in compensating for the low kinase activity and defining the early CDK thresholds. These fine-tuned docking interactions help to achieve temporal order and localization specificity of early cell cycle events via CDK thresholds and are important for avoiding errors, and thereby, ensuring better cellular fitness and efficient cell proliferation.

The quantitative analysis of a subset of G1-specific CDK targets revealed that besides the known LP docking motif, the G1-CDKs use yet other unknown docking mechanisms. We identified a basic potentially helical docking motif, denoted as K/R motif that was present in several early CDK targets. Next, we described Cln2 mutants that were incapable of this interaction, enabling us to identify more K/R docking dependent G1-Cdk1 substrates. Even though the K/R motif was found to be critical in efficient phosphorylation of a range of G1-Cdk1 targets, further studies are needed to evaluate the importance of this docking mechanism in vivo. Our results are in a line with the prediction from a recent study reporting that the G1 cyclins of *S. cerevisiae* have a specific function in promoting polarization and growth of the buds, while the known G1-CDK specific LP docking motif was not responsible for this function [30]. The study concluded that Cln2 plays a unique role during bud formation by phosphorylating targets in the cell polarity and budding pathways that are not phosphorylated by mitotic Clb2-Cdk complexes. The authors showed that after the release from pheromone induced arrest the cells that expressed G1 cyclin Cln2 retained shmoo tip induced polarity at the sites of bud formation, while the polarization was lost in cells lacking Cln2, leaving actin in patches dispersed around the cell. Previous studies have shown that polarized growth proteins Boi1, Rga2 and Cdc24, are more efficiently targeted by Cln2-Cdk in vitro and in vivo [38]. In the present study, we found that one of them, a guanine nucleotide exchange factor Cdc24 is phosphorylated in the early cell cycle, while the phosphorylation is considerably decreased in cells expressing Cln2 K/R docking mutants. In addition, several other relevant CDK targets contain candidate K/R motifs (Figure S5). However, the Cln2 mutants deficient in either LP or K/R docking were capable of driving cell cycle progression as the only G1 cyclin indicating that these docking interactions are not essential for the G1 function. Therefore, it is possible that yet other G1-CDK specific substrate recognition mechanisms remain to be discovered or that presence of either LP or K/R docking is sufficient to carry out the essential function of G1 cyclins. Alternatively, this function of G1-CDK could be independent of cyclin docking, but instead could be mediated by the cyclin-specificity of CDK inhibitor Sic1 [44] or more precise dosing of CDK activity that depends on the increasing intrinsic activity of sequential cyclin-CDK complexes might be critical [11]. Indeed, in our screens on selected targets, we have found that Whi7 is a G1-CDK specific target, whose phosphorylation is not affected by K/R or LP mutations. In addition, one Cln1,2 specificity criteria lies within the phosphorylation site consensus motif. The Clb complexes are insensitive to positive amino acids in position +2 from the phosphorylated residue, while the Cln1,2 complexes use +2K/R as a strong specificity element [11].

Interestingly, a recent study reported that the human cyclin D-Cdk4,6 complex also docks one side of an α-helix in the Rb C terminus, which is not recognized by cyclins E, A and B [12]. These findings combined with the current study on yeast G1-CDK complexes suggest that helical docking modules may be a common evolutionarily conserved mechanism to facilitate substrate recognition by CDK. Even more interesting is the fact that both studies have found such docking is the case of early G1 CDK complexes. More studies are needed to clarify the extent of the general importance of these novel mechanisms that differ from the conventional concept of short linear docking motifs (SLiMs) used for cyclin docking and kinase docking in general [45], that in most cases are considered not to involve or depend on secondary structure elements.

An extra unique feature of the discovered docking mechanism was the finding that the K/R docking modules can be switched off by phosphorylation with PKA. This result was only demonstrated in vitro and should be further validated using a cell-based assay with an inducible PKA signal [43]. Nevertheless, such mechanism with two kinase inputs, where one is modulating the substrate docking efficiency of the other via phosphorylation of a distant docking motif is very attractive and unique, and it is worth considering as a modular element for synthetic biology applications in the area of de novo design of protein signaling networks.

In conclusion, the study expands the diversity of known cyclin-based protein docking mechanisms and sheds light on the recently discovered essential function of G1-cyclin specificity mechanisms in *S. cerevisiae*. A growing list of different docking motifs widens the combinatorial toolbox of modules recognized by the CDK complex that can potentially be used to flexibly encode different CDK thresholds.

## 4. Materials and Methods

### 4.1. Yeast Strains

The yeast strains used in the study were haploid derivates of W303 background and are described in Table S1. PCR and homologous recombination based methods were used for promoter substitutions, gene deletions and tagging [46,47]. Cloning was performed with PCR, restriction enzyme digestion and ligation. All constructs and gene modifications were verified by DNA sequencing. Cln2 mutants were expressed from pRS314-based vector containing 1–1000 base pairs upstream of *CLN2* reading frame as the *CLN2* promoter and a C-terminal 7myc tag.

### 4.2. Protein Purification

GST-tagged substrate proteins were expressed from pGEX-4T-1 and 6xHis-tagged proteins from pET28a vectors in *E. coli* BL21RP cells. The vectors used for protein expression are listed in Table S2. Sic1, Nrm1, Sli15 and Bud2 were expressed at 37 °C using 1 mM IPTG. Expression of other proteins was induced with 0.3 mM IPTG at 18 °C for 16 h. Cells were lysed using lysozyme and GST-tagged proteins were purified using glutathione-Sepharose beads (GE Healthcare, Chicago, IL, USA). Standard immobilized cobalt affinity purification and elution with imidazole was used for purification of His-tagged proteins.

Clb5-, Clb3- and Clb2-Cdk1 were purified from yeast cells using TAP-tagged cyclins as described previously [48,49]. Cln2-Cdk1 complex was purified using N-terminally 3HA-tagged Cln2 and immunoaffinity chromatography with antibody against HA epitope as described previously [38]. Purification of different Cln2 mutants was carried out by expressing GST-Cln2 from pRS413-based vectors. For purification, expression of the cyclins was induced from GAL1 promoter and the cells were lysed using Mixer Mill MM 400 (Retsch, Haan, Germany). Recombinant Cks1 was purified from *E. coli* BL21RP as described previously [50].

### 4.3. Kinase Assay

The phosphorylation reactions were carried out at room temperature in buffer containing 50 mM Hepes-KOH, pH 7.4, 150 mM NaCl, 5 mM MgCl_2_, 20 mM imidazole, 2.5 mM L-glutathione, 2% glycerol, 0.2 mg/mL BSA, 500 nM Cks1 and 500 µM ATP [(with added [γ-^32^P]-ATP (Hartmann Analytic, Braunschweig, Germany)]. Substrate protein concentrations were 0.5–2 µM (in the linear [S] versus v0 range, several-fold below the estimated K_M_ value). The concentrations of kinase complexes were around 0.2–2 nM. The kinase assays were performed under conditions below 10% of initial substrate turnover. To study the effect of Bud2ΔN pre-phosphorylation by PKA on the K/R docking, 50 µM Bud2 was first phosphorylated with 2 µM PKA for 30 min, followed by purification of GST-Bud2ΔN using glutathione agarose affinity chromatography. Phosphorylation reactions were stopped with SDS-PAGE sample buffer and separated using SDS-PAGE.

γ-^32^P phosphorylation signals were detected using an Amersham Typhoon 5 Biomolecular Imager (GE Healthcare Life Sciences, Chicago, IL, USA). Signals were quantified using ImageQuant TL (Amersham Biosciences, Amersham, UK), and GraphPad Prism 8 (San Diego, CA, USA) was used for data analysis. All kinase assays were performed in at least two replicate experiments.

### 4.4. Elongated Growth Assay

Yeast cells containing different pRS314-*P_GAL1_-GST-CLN2* plasmids were grown at 30 °C in synthetic complete medium lacking histidine supplemented with 2% raffinose. At OD_600_=0.5, GST-Cln2 overexpression was induced by addition of 2% galactose. 2 h after induction the cells were fixed with formaldehyde. The cells were imaged using Axio Observer.Z1 microscope (Zeiss, Oberkochen, Germany) with 63×/1.4 objective. For every Cln2 variant, 90 buds were analyzed. The bud length from the bud neck to the tip was measured in ImageJ.

### 4.5. Microscopy

Time-lapse microscopy was used to measure the time of budding from Start. Cells were grown at 30 °C in synthetic complete media with 2% glucose (SC) to OD_600_ 0.2–0.6 before the experiment. Cells were then pipetted onto 0.8-mm cover glass and covered with a 1-mm thick 2% SC/glucose agarose pad (NuSieve™ GTG™ Agarose, Lonza, Basel, Switzerland). Cells were incubated under the agarose pad for 1 h before the start of the experiment. Imaging was executed using a Zeiss Observer Z1 microscope with a 63×/1.4NA oil immersion objective and Orca-r2 C10600-10B camera (Hamamatsu Photonics, Hamamatsu, Japan), using 3 × 3 binning. The sample was kept at 30 °C using Tempcontrol 37-2 digital (PeCon, Erbach, Germany). Cells were imaged every 3 min in 8 h long experiments and contained up to 12 positions that were followed using an automated stage and ZEN software (Zeiss). Focus was kept using Definite Focus. Start point was defined by the nuclear Whi5-mCherry level dropping to 50% of the G1 level [51].

To study the localization of Sli15 and the spindle dynamics, cells expressing Sli15-EGFP and Whi5-mCherry or Spc42-EGFP and Tub1-mCherry were grown to OD_600_ 0.2–0.6, pipetted onto the 0.8 mm cover glass and covered with 1 mm thick 2% SC/glucose agarose pad (NuSieve™ GTG™ Agarose, Lonza). Colibri 470 LED module with exposure time of 15 ms and 150 ms was used for excitation of Spc42-EGFP and Sli15-EGFP, respectively. Whi5-mCherry and Tub1-mCherry were excited using Colibri 540–580 LED module for 750 ms. All Colibri modules were used at 25% power. Filter set 61 HE (Zeiss) was used for imaging EGFP and mCherry. To study the localization of Sli15-EGFP, z-stack images at 5 focal planes were taken.

Image segmentation, cell tracking and quantification of fluorescence signals were performed using MATLAB (The MathWorks, Inc., Natick, MA, USA) as described in [52]. All plots with microscopy data contain data from at least two experiments.

### 4.6. Western Blot

For cell cycle synchronization, the cultures were grown to OD_600_ = 0.3 at 30 °C, followed by addition of α-factor at final concentration of 1 mg/L. The cultures were grown for 2.5 h, when the cells were pelleted, washed thoroughly and resuspended in medium without α-factor to release the cells to the cell cycle. At the indicated time point, cells were pelleted and snap-frozen. The cells were resuspended in urea lysis buffer and were lysed by bead beating. After clearing the lysate by centrifugation, the total protein concentration was measured with Bio-Rad Protein Assay Dye Reagent. Cln2-7myc and Whi7-13myc samples were loaded on 8% acrylamide SDS-PAGE gels. Cdc24-13myc phosphorylation was analyzed using 6% acrylamide Mn^2+^-Phos-tag SDS-PAGE gels supplemented with 25 µM Phos-tag [53]. The proteins were transferred to nitrocellulose membranes using Pierce G2 Fast Blotter (ThermoFisher Scientific, Waltham, MA, USA). For loading control, the membranes were stained with Ponceau S dye. Rabbit polyclonal c-myc antibody (sc-789, Santa Cruz Biotechology, Dallas, TX, USA) and HRP-conjugated anti-rabbit IgG antibody (1:7500) from Labas, Estonia were used for detection of myc-tagged proteins.

### 4.7. Serial Dilutions Growth Assay

The *cln1*Δ *cln2*Δ *P_GAL1_-3HA-CLN3* yeast strain was transformed with plasmids expressing different Cln2 mutants. The strains were grown in synthetic complete medium lacking tryptophan supplemented with 2% raffinose and 2% galactose to OD_600_ = 0.2–0.6. The cultures were diluted to 10^6^ cells/mL, followed by dilution by the factor of 2, 10, 100 and 500 times. 5 µL of the dilutions was plated on synthetic complete medium lacking tryptophan with either 2% glucose to suppress *P_GAL1_-3HA-CLN3* expression or 2% raffinose and 2% galactose. The plates were incubated at 30 °C for 48 h. The experiment was repeated twice.

### 4.8. Bioinformatics

Protein secondary structure predictions were performed using PSIPRED [54]. SlimSearch was used to search for potential K/R motifs in Cdk1 targets [55]. Cln2-Cdk2-Cks1 structure was created based on cyclin A-Cdk2-Cks1 model [27] using I-TASSER to model the structure of Cln2 and Tm-align to combine the Cln2 structure with Cdk2-Cks1 structure [56,57].

## Data Availability

The data presented in this study are available in Faustova, I.; Möll, K.; Valk, E.; Loog, M.; Örd, M. Docking to a Basic Helix Promotes Specific Phosphorylation by G1-Cdk1. *Int. J. Mol. Sci.* **2021**, *22*, 9514. https://doi.org/10.3390/ijms22179514.

## Supplementary Materials

The following are available online at https://www.mdpi.com/article/10.3390/ijms22179514/s1.

## Abbreviations

CDK	cyclin-dependent kinase
SLiM	short linear motif
SPB	spindle pole body
PKA	protein kinase A

## References

[1] Morgan D.O. (2007). The Cell Cycle: Principles of Control.

[2] Enserink J.M., Kolodner R.D. (2010). An overview of Cdk1-controlled targets and processes. Cell Div..

[3] Stern B., Nurse P. (1996). A quantitative model for the cdc2 control of S phase and mitosis in fission yeast. Trends Genet..

[4] Swaffer M.P., Jones A.W., Flynn H.R., Snijders A.P., Nurse P. (2016). CDK Substrate Phosphorylation and Ordering the Cell Cycle. Cell.

[5] Fisher D.L., Nurse P. (1996). A single fission yeast mitotic cyclin B p34cdc2 kinase promotes both S-phase and mitosis in the absence of G1 cyclins. EMBO J..

[6] Coudreuse D., Nurse P. (2010). Driving the cell cycle with a minimal CDK control network. Nature.

[7] Örd M., Loog M. (2019). How the cell cycle clock ticks. Mol. Biol. Cell.

[8] Bloom J., Cross F.R. (2007). Multiple levels of cyclin specificity in cell-cycle control. Nat. Rev. Mol. Cell Biol..

[9] Crncec A., Hochegger H. (2019). Triggering mitosis. FEBS Lett..

[10] Tatum N.J., Endicott J.A. (2020). Chatterboxes: The structural and functional diversity of cyclins. Semin. Cell Dev. Biol..

[11] Kõivomägi M., Valk E., Venta R., Iofik A., Lepiku M., Morgan D.O., Loog M. (2011). Dynamics of Cdk1 Substrate Specificity during the Cell Cycle. Mol. Cell.

[12] Topacio B.R., Zatulovskiy E., Cristea S., Xie S., Tambo C.S., Rubin S.M., Sage J., Kõivomägi M., Skotheim J.M. (2019). Cyclin D-Cdk4,6 Drives Cell-Cycle Progression via the Retinoblastoma Protein’s C-Terminal Helix. Mol. Cell.

[13] Bhaduri S., Pryciak P.M. (2011). Cyclin-specific docking motifs promote phosphorylation of yeast signaling proteins by G1/S Cdk complexes. Curr. Biol..

[14] Bandyopadhyay S., Bhaduri S., Örd M., Davey N.E., Loog M., Pryciak P.M. (2020). Comprehensive Analysis of G1 Cyclin Docking Motif Sequences that Control CDK Regulatory Potency In Vivo. Curr. Biol..

[15] Schulman B.A., Lindstrom D.L., Harlow E.D. (1998). Substrate recruitment to cyclin-dependent kinase 2 by a multipurpose docking site on cyclin A. Proc. Natl. Acad. Sci. USA.

[16] Chen J., Saha P., Kornbluth S., Dynlacht B.D., Dutta A. (1996). Cyclin-binding motifs are essential for the function of p21CIP1. Mol. Cell. Biol..

[17] Wilmes G.M., Archambault V., Austin R.J., Jacobson M.D., Bell S.P., Cross F.R. (2004). Interaction of the S-phase cyclin Clb5 with an RXL docking sequence in the initiator protein Orc6 provides an origin-localized replication control switch. Genes Dev..

[18] Loog M., Morgan D.O. (2005). Cyclin specificity in the phosphorylation of cyclin-dependent kinase substrates. Nature.

[19] Faustova I., Bulatovic L., Matiyevskaya F., Valk E., Örd M., Loog M. (2020). A new linear cyclin docking motif that mediates exclusively S-phase CDK-specific signaling. EMBO J..

[20] Örd M., Puss K.K., Kivi R., Möll K., Ojala T., Borovko I., Faustova I., Venta R., Valk E., Kõivomägi M. (2020). Proline-Rich Motifs Control G2-CDK Target Phosphorylation and Priming an Anchoring Protein for Polo Kinase Localization. Cell Rep..

[21] Örd M., Venta R., Möll K., Valk E., Loog M. (2019). Cyclin-Specific Docking Mechanisms Reveal the Complexity of M-CDK Function in the Cell Cycle. Mol. Cell.

[22] Bremmer S.C., Hall H., Martinez J.S., Eissler C.L., Hinrichsen T.H., Rossie S., Parker L.L., Hall M.C., Charbonneau H. (2012). Cdc14 phosphatases preferentially dephosphorylate a subset of cyclin-dependent kinase (Cdk) sites containing phosphoserine. J. Biol. Chem..

[23] Godfrey M., Touati S.A., Kataria M., Jones A., Snijders A.P., Uhlmann F. (2017). PP2ACdc55 Phosphatase Imposes Ordered Cell-Cycle Phosphorylation by Opposing Threonine Phosphorylation. Mol. Cell.

[24] Touati S.A., Hofbauer L., Jones A.W., Snijders A.P., Kelly G., Uhlmann F. (2019). Cdc14 and PP2A Phosphatases Cooperate to Shape Phosphoproteome Dynamics during Mitotic Exit. Cell Rep..

[25] Kataria M., Mouilleron S., Seo M.-H., Corbi-Verge C., Kim P.M., Uhlmann F. (2018). A PxL motif promotes timely cell cycle substrate dephosphorylation by the Cdc14 phosphatase. Nat. Struct. Mol. Biol..

[26] McGrath D.A., Balog E.R.M., Kõivomägi M., Lucena R., Mai M.V., Hirschi A., Kellogg D.R., Loog M., Rubin S.M. (2013). Cks confers specificity to phosphorylation-dependent CDK signaling pathways. Nat. Struct. Mol. Biol..

[27] Kõivomägi M., Örd M., Iofik A., Valk E., Venta R., Faustova I., Kivi R., Balog E.R.M., Rubin S.M., Loog M. (2013). Multisite phosphorylation networks as signal processors for Cdk1. Nat. Struct. Mol. Biol..

[28] Örd M., Möll K., Agerova A., Kivi R., Faustova I., Venta R., Valk E., Loog M. (2019). Multisite phosphorylation code of CDK. Nat. Struct. Mol. Biol..

[29] Holt L.J., Tuch B.B., Villén J., Johnson A.D., Gygi S.P., Morgan D.O. (2009). Global analysis of Cdk1 substrate phosphorylation sites provides insights into evolution. Science.

[30] Ercan D.P., Chrétien F., Chakravarty P., Flynn H.R., Snijders A.P., Uhlmann F. (2021). Budding yeast relies on G1 cyclin specificity to couple cell cycle progression with morphogenetic development. Sci. Adv..

[31] Bhaduri S., Valk E., Winters M.J., Gruessner B., Loog M., Pryciak P.M. (2015). A docking interface in the cyclin Cln2 promotes multi-site phosphorylation of substrates and timely cell-cycle entry. Curr. Biol..

[32] Songyang Z., Blechner S., Hoagland N., Hoekstra M.F., Piwnica-Worms H., Cantley L.C. (1994). Use of an oriented peptide library to determine the optimal substrates of protein kinases. Curr. Biol..

[33] Suzuki K., Sako K., Akiyama K., Isoda M., Senoo C., Nakajo N., Sagata N. (2015). Identification of non-Ser/Thr-Pro consensus motifs for Cdk1 and their roles in mitotic regulation of C2H2 zinc finger proteins and Ect2. Sci. Rep..

[34] Chant J., Herskowitz I. (1991). Genetic control of bud site selection in yeast by a set of gene products that constitute a morphogenetic pathway. Cell.

[35] Schneider J., Bajwa P., Johnson F., Bhaumik S., Shilatifard A. (2006). Rtt109 is required for proper H3K56 acetylation: A chromatin mark associated with the elongating RNA polymerase II. J. Biol. Chem..

[36] Yahya G., Parisi E., Flores A., Gallego C., Aldea M. (2014). A Whi7-Anchored Loop Controls the G1 Cdk-Cyclin Complex at Start. Mol. Cell.

[37] Lew D.J., Reed S.I. (1993). Morphogenesis in the yeast cell cycle: Regulation by Cdc28 and cyclins. J. Cell Biol..

[38] McCusker D., Denison C., Anderson S., Egelhofer T.A., Yates J.R., Gygi S.P., Kellogg D.R. (2007). Cdk1 coordinates cell-surface growth with the cell cycle. Nat. Cell Biol..

[39] Jaspersen S.L., Huneycutt B.J., Giddings T.H., Resing K.A., Ahn N.G., Winey M. (2004). Cdc28/Cdk1 Regulates Spindle Pole Body Duplication through Phosphorylation of Spc42 and Mps1. Dev. Cell.

[40] Crasta K., Huang P., Morgan G., Winey M., Surana U. (2006). Cdk1 regulates centrosome separation by restraining proteolysis of microtubule-associated proteins. EMBO J..

[41] Elserafy M., Šarić M., Neuner A., Lin T.C., Zhang W., Seybold C., Sivashanmugam L., Schiebel E. (2014). Molecular mechanisms that restrict yeast centrosome duplication to one event per cell cycle. Curr. Biol..

[42] Elbaum M., Zondlo N. (2014). OGlcNAcylation and phosphorylation have similar structural effects in α-helices: Post-translational modifications as inducible start and stop signals in α-helices, with greater structural effects on threonine modification. Biochemistry.

[43] Amigoni L., Colombo S., Belotti F., Alberghina L., Martegani E. (2015). The transcription factor Swi4 is target for PKA regulation of cell size at the G1 to S transition in Saccharomyces cerevisiae. Cell Cycle.

[44] Tyers M. (1996). The cyclin-dependent kinase inhibitor p40SIC1 imposes the requirement for Cln G1 cyclin function at Start. Proc. Natl. Acad. Sci. USA.

[45] Tompa P., Davey N.E., Gibson T.J., Babu M.M. (2014). A Million Peptide Motifs for the Molecular Biologist. Mol. Cell.

[46] Longtine M.S., Mckenzie A., Demarini D.J., Shah N.G., Wach A., Brachat A., Philippsen P., Pringle J.R. (1998). Additional modules for versatile and economical PCR-based gene deletion and modification in Saccharomyces cerevisiae. Yeast.

[47] Janke C., Magiera M.M., Rathfelder N., Taxis C., Reber S., Maekawa H., Moreno-Borchart A., Doenges G., Schwob E., Schiebel E. (2004). A versatile toolbox for PCR-based tagging of yeast genes: New fluorescent proteins, more markers and promoter substitution cassettes. Yeast.

[48] Puig O., Caspary F., Rigaut G., Rutz B., Bouveret E., Bragado-Nilsson E., Wilm M., Séraphin B. (2001). The Tandem Affinity Purification (TAP) Method: A General Procedure of Protein Complex Purification. Methods.

[49] Ubersax J.A., Woodbury E.L., Quang P.N., Paraz M., Blethrow J.D., Shah K., Shokat K.M., Morgan D.O. (2003). Targets of the cyclin-dependent kinase Cdk1. Nature.

[50] Reynard G.J., Reynolds W., Verma R., Deshaies R.J. (2000). Cks1 is required for G(1) cyclin-cyclin-dependent kinase activity in budding yeast. Mol. Cell. Biol..

[51] Doncic A., Falleur-Fettig M., Skotheim J.M. (2011). Distinct Interactions Select and Maintain a Specific Cell Fate. Mol. Cell.

[52] Doncic A., Eser U., Atay O., Skotheim J.M. (2013). An algorithm to automate yeast segmentation and tracking. PLoS ONE.

[53] Örd M., Loog M. (2020). Detection of Multisite Phosphorylation of Intrinsically Disordered Proteins Using Phos-tag SDS-PAGE. Methods Mol. Biol..

[54] Buchan D.W.A., Minneci F., Nugent T.C.O., Bryson K., Jones D.T. (2013). Scalable web services for the PSIPRED Protein Analysis Workbench. Nucleic Acids Res..

[55] Krystkowiak I., Davey N.E. (2017). SLiMSearch: A framework for proteome-wide discovery and annotation of functional modules in intrinsically disordered regions. Nucleic Acids Res..

[56] Yang J., Yan R., Roy A., Xu D., Poisson J., Zhang Y. (2014). The I-TASSER suite: Protein structure and function prediction. Nat. Methods.

[57] Zhang Y., Skolnick J. (2005). TM-align: A protein structure alignment algorithm based on the TM-score. Nucleic Acids Res..

