# Supplementary material for: Docking to a Basic Helix Promotes Specific Phosphorylation by G1-Cdk1
Source: Int J Mol Sci. 2021 Sep 1;22(17):9514. doi: 10.3390/ijms22179514 (PMC8431026; doi:10.3390/ijms22179514)
Supplement: Supplementary file 1 [file ijms-22-09514-s001.zip › ijms-1317427-supplementary.pdf]

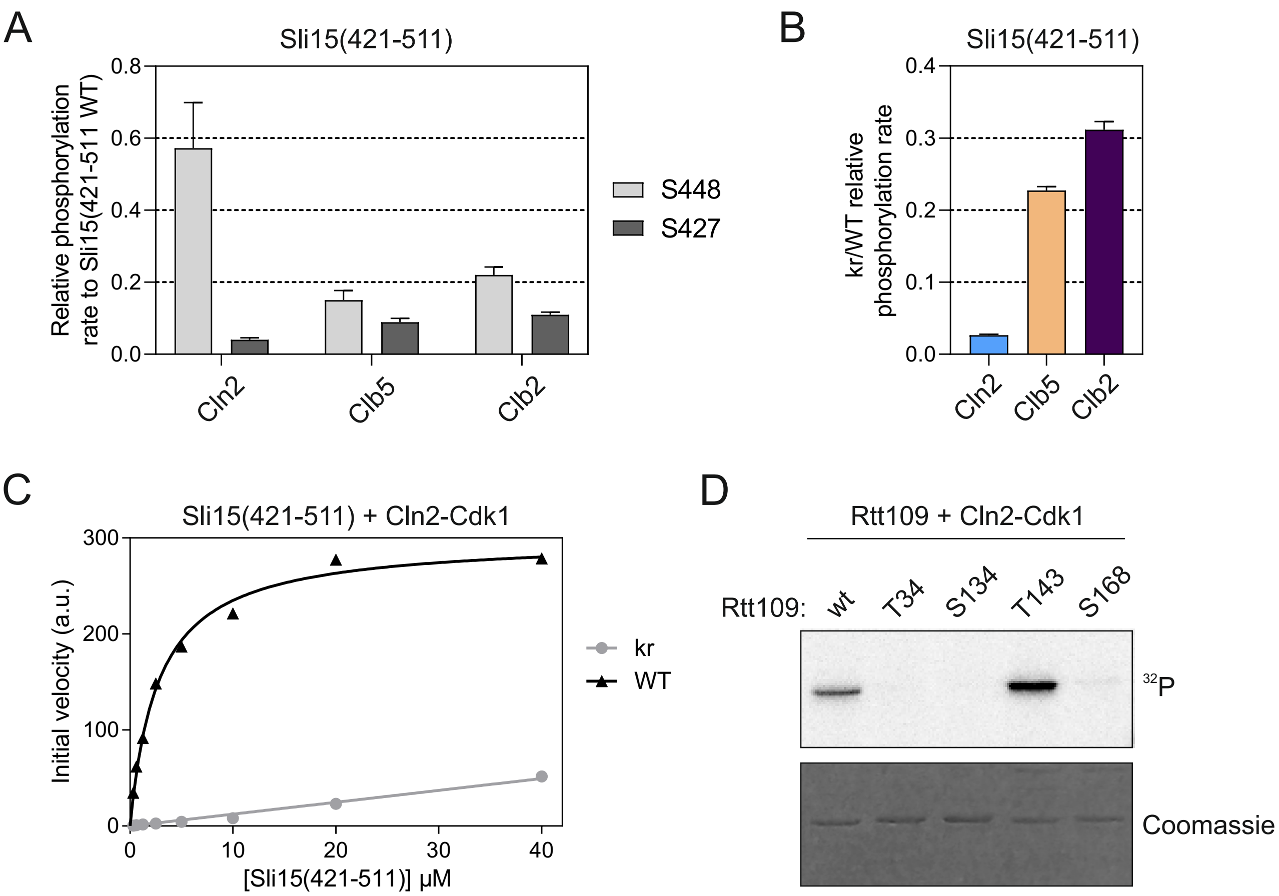

Figure S1. K/R motifs promote phosphorylation by Cln2-Cdk1. (**A**) Plot showing the relative phosphorylation rate of Sli15(421-511) S427 or S448 to Sli15(421-511) with all Cdk1 consensus sites by Cln2-, Clb5- and Clb2-Cdk1. The error bars show standard deviation of the mean of replicate experiments. (**B**) The relative phosphorylation rate of wild-type to the KR docking mutant Sli15(421-511) by different Cdk1 complexes. The error bars show standard deviation. (**C**) Michaelis-Menten curves of the phosphorylation of wild-type or KR docking mutant Sli15(421-511) by Cln2-Cdk1. This experiment was performed once. (**D**) ^{32}P autoradiograph showing Cln2-Cdk1-mediated phosphorylation of wild-type Rtt109 or its mutants containing only the indicated CDK consensus phosphorylation site. Below, the Coomassie-stained gel showing the amount of Rtt109 in the assay is shown.

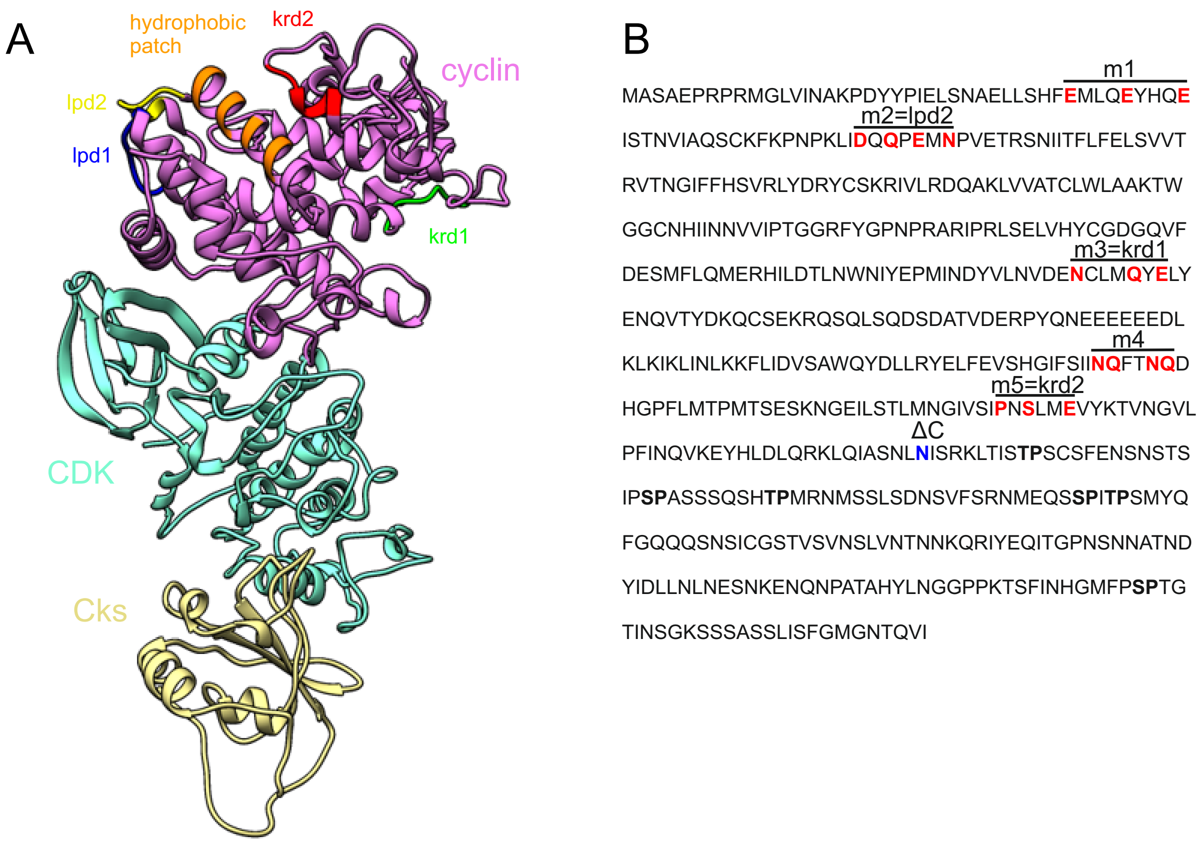

Figure S2. Cln2 mutants used in the study. (**A**) Structural model of Cln2-CDK-Cks complex showing the position of the mutations used in the study. lpd1 denotes the previously identified LP docking pocket (Bhaduri *et al*., 2015). The structural model was created based on cyclin A-Cdk2-Cks1 model (Kõivomägi *et al*., 2013) by replacing cyclin A with Cln2 using I-TASSER and TM-align (Yang *et al*., 2014; Zhang *et al*., 2005). (**B**) Sequence of Cln2 with the residues mutated to alanine in the indicated Cln2 mutant shown in red. Cln1(m1) is E35A E39A E43A mutant, Cln2(m2, lpd2) is D63A Q65A E67A N69A mutant, Cln2(m3, krd1) is N209A Q213A E215A mutant, Cln2(m4) is N298A Q299A N302A Q303A mutant, Cln2(m5, krd2) is P334A S336A E339A mutant. The Cln2 mutants lacking the intrinsically disordered C terminus contain Cln2 residues 1-372.

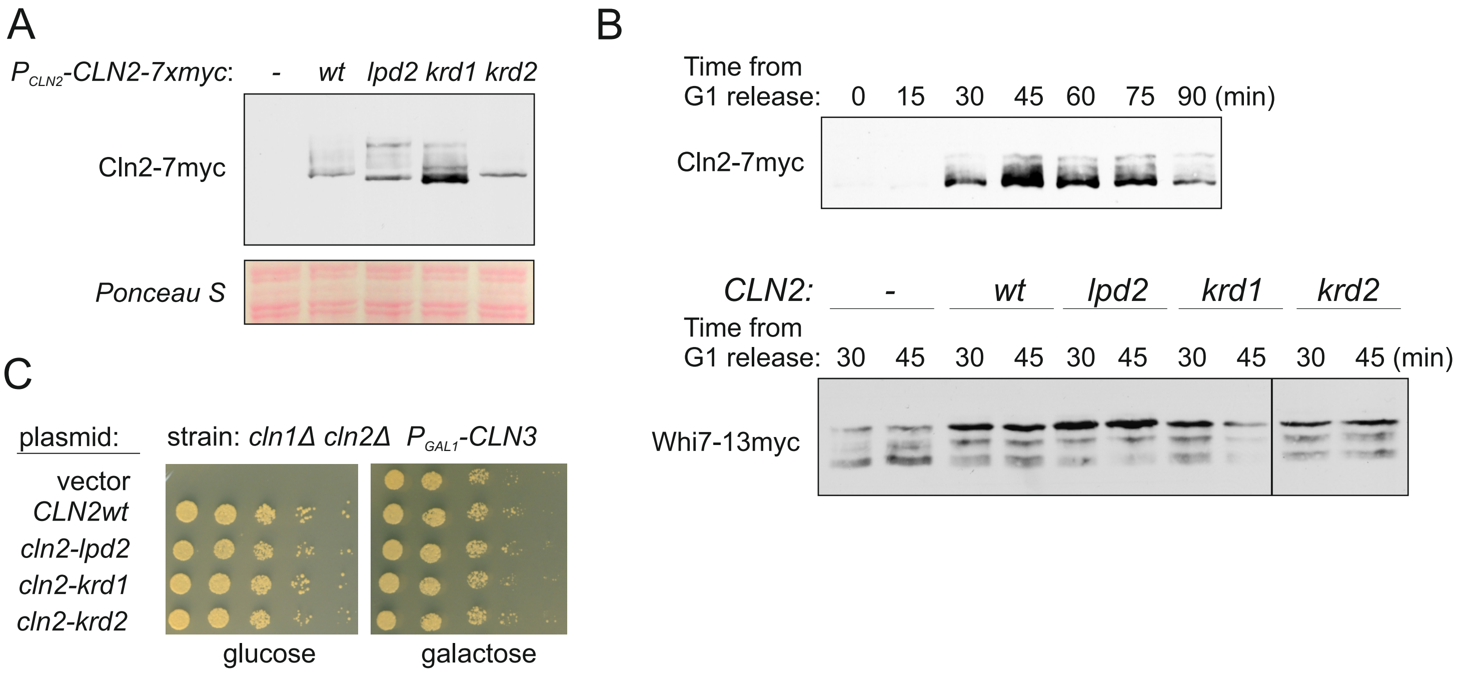

Figure S3. The expression and functionality of Cln2 mutants. (**A**) The expression of different Cln2 mutants in asynchronous cultures was studied by western blot. *Ponceau S* stains total protein. (**B**) The expression profile of Cln2(wt)-7myc in synchronized culture released from α-factor induced G1 arrest (top). The phosphorylation of Whi7-13myc was studied at 30 and 45 minutes after release from G1 arrest in cells expressing different Cln2 mutants from *CLN2* promoter in *cln1Δ* strain (bottom). (**C**) Serial dilution growth assay showing the rescue of cln deletion by all tested Cln2 mutants. The Cln2 mutants were expressed from *CLN2* promoter on a centromeric plasmid.

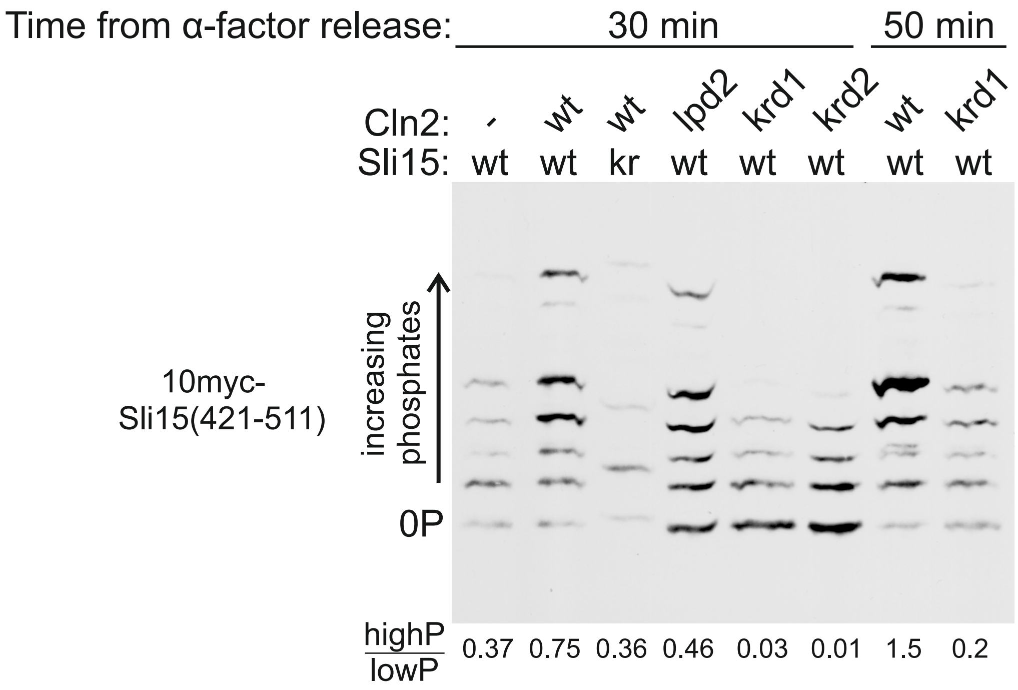

Figure S4. Multisite phosphorylation analysis of the Sli15(421-511) fragment *in vivo*. Cells of different *cln1Δ* strains expressing the indicated Cln2 (from *CLN2* promoter) and 10myc-Sli15(421-511) (from ADH1 promoter) mutants were arrested in G1 with pheromone and were released to the cell cycle. Multisite phosphorylation of the Sli15 fragment was studied 30 minutes after release from G1 arrest using Phos-tag SDS-PAGE western blot.

Sli15(477-491): RLKIKEKTLRKLSPN
Boi2(686-704): VDKKSSKKSRSKRRSVSAK
Fus1(395-409): KPLKKRKKRRQSKMY
Sac7(290-308): DEEKRRRKIRHKRRLTRDI
Bnr1(183-203): GMKLSKALYKLEKFLRKQSFL
Sfh1(385-408): KEEIQKREIEKERNLRRLKRETDR
Bud3(361-380): AATKNFSKDIKLFIRHFSNV

Figure S5. Negative regulation of K/R docking via phosphorylation by PKA. Sequences of predicted K/R motifs that overlap with PKA phosphorylation sites. The basic residues that constitute the K/R motif are in red, predicted PKA site in blue, and predicted α-helixes are highlighted in yellow.

Table S1. Yeast strains used in this study. All strains are in W303 background.

Strain	Genotype	Source
DMY305	*bar1Δ cln2::TRP1::GAL1-3HA-CLN2*	McCusker et al., 2007
DOM0076	*bar1Δ::HISG sic1Δ::LEU2 pRS426-GAL1-CLB5-TAP*	Ubersax et al., 2003
DOM0077	*bar1Δ::HISG sic1Δ::LEU2 pRS426-GAL1-CLB2-TAP*	Ubersax et al., 2003
DOM0957	*bar1Δ::HISG sic1Δ::LEU2 pRS426-GAL1-CLB3-TAP*	Kõivomägi et al., 2011
PPY2444	*bar1Δ cln2::TRP1::GAL1-3HA-CLN2(lpd)*	Bhaduri et al., 2015
DK252	*MAT***a** *URA3::* P_{GAL1}*-CLN3 cln1Δ::HIS3 cln2Δ::LEU2 bar1Δ::hisG*	Egelhofer et al., 2008
MO038	*MAT***a** *cln1Δ::LEU2 cln2Δ::URA3 bar1Δ::hisG*	This study
MO040	*MAT***a** *cln1Δ::LEU2 cln2Δ::URA3 WHI5-mCherry::HIS3 SIC1-EGFP::kanMX4 bar1Δ::hisG*	This study
MO047	*MAT***a** *cln1Δ::LEU2 cln2Δ::URA3 WHI5-mCherry::HIS3 SLI15-EGFP::kanMX4 bar1Δ::hisG*	This study
MO061	*MAT***a** *cln1Δ::LEU2 cln2Δ::URA3 WHI7-13MYC::kanMX4 bar1Δ::hisG*	This study
MO062	*MAT***a** *cln1Δ::LEU2 cln2Δ::URA3 CDC24-13MYC::kanMX4 bar1Δ::hisG*	This study
MO069	*MAT***a** *cln1Δ::LEU2 cln2Δ::URA3 SPC42-EGFP::kanMX4 his3::HIS3::*P_{CYC1}*-mCherry-TUB1 bar1Δ::hisG*	This study

Table S2. Plasmids used in the study.

Name	Backbone	Description	Source
pMO013	pGEX-4T-1	GST-Sli15(422-521)	This study
pMO014	pGEX-4T-1	GST-Sli15(422-511)	This study
pMO016	pGEX-4T-1	GST-Sli15(422-511; S448)	This study
pMO025	pGEX-4T-1	GST-Sli15(422-511; S427)	This study
pMO030	pET28a	6xHis-Rtt109(S168)	This study
pMO031	pET28a	6xHis-Rtt109(T34)	This study
pMO032	pET28a	6xHis-Rtt109(T143)	This study
pMO035	pGEX-4T-1	GST-Sli15(422-454; S448)	This study
pMO042	pGEX-4T-1	GST-Sli15(422-497; S448)	This study
pMO043	pGEX-4T-1	GST-Sli15(422-483; S448)	This study
pMO044	pGEX-4T-1	GST-Sli15(422-470; S448)	This study
pMO056	pGEX-4T-1	GST-Sli15(422-511; S448 RKSK-AASA)	This study
pMO061	pGEX-4T-1	GST-Sli15(422-511; S448 K496A)	This study
pMO062	pGEX-4T-1	GST-Sli15(422-511; S448 KK485AA)	This study
pMO067	pGEX-4T-1	GST-Sli15(422-511; S448 KNK-ANA)	This study
pMO068	pGEX-4T-1	GST-Sli15(422-511 S448; RLKIKEK-ALAIAEA)	This study
pMO071	pET28a	6xHis-Rtt109(RRLK-AALA(h1))	This study
pMO075	pGEX-4T-1	GST-Sli15(422-511; S448 rk)	This study
pMO076	pET28a	6xHis-Rtt109(S134)	This study
pMO081	pET28a	6xHis-Rtt109(RKQFRAIKK-AAQFAAIAA(h2))	This study
pPP2154	pRS413	CEN *HIS3* P_{GAL1}*-GST* T_{CYC1}	Bhaduri and Pryciak, 2011
pPP3572	pRS413	CEN *HIS3* P_{GAL1}*-GST-CLN2* T_{CYC1}	Bhaduri and Pryciak, 2011
pMO103	pRS413	CEN *HIS3* P_{GAL1}*-GST-CLN2(m1)* T_{CYC1}	This study
pMO109	pRS413	CEN *HIS3* P_{GAL1}*-GST-CLN2(m4)* T_{CYC1}	This study
pMO110	pRS413	CEN *HIS3* P_{GAL1}*-GST-CLN2(m2)* T_{CYC1}	This study
pMO111	pRS413	CEN *HIS3* P_{GAL1}*-GST-CLN2(1-362)* T_{CYC1}	This study

pMO121	pGEX-4T-1	GST-Bud2(1001-1104)	This study
pMO122	pET28a	6xHis-Ste7	This study
pMO123	pRS413	CEN *HIS3* P_{GAL1}-*GST-CLN2(m3)* T_{CYC1}	This study
pMO124	pRS413	CEN *HIS3* P_{GAL1}-*GST-CLN2(m5)* T_{CYC1}	This study
pMO128	pGEX-4T-1	GST-Bud2(1001-1104 h-mut)	This study
pMO130	pGEX-4T-1	GST-Spc29	This study
pMO164	pRS313	P_{ADH} *10MYC-SLI15(422-511)*	This study
pMO173	pRS314	P_{CLN2} *CLN2(wt)-7MYC*	This study
pMO174	pRS314	P_{CLN2} *CLN2(krd2)-7MYC*	This study
pMO178	pRS314	P_{CLN2} *7MYC*	This study
pMO179	pRS314	P_{CLN2} *CLN2(krd1)-7MYC*	This study
pMO180	pRS314	P_{CLN2} *CLN2(lpd2)-7MYC*	This study
pMO183	pRS314	P_{CLN2}	This study
pMO184	pRS314	P_{CLN2} *CLN2(wt)*	This study
pMO185	pRS314	P_{CLN2} *CLN2(lpd2)*	This study
pMO186	pRS314	P_{CLN2} *CLN2(krd1)*	This study
pMO187	pRS314	P_{CLN2} *CLN2(krd2)*	This study
pMO189	pRS413	CEN *HIS3* P_{GAL1}-*GST-CLN2(6A)* T_{CYC1}	This study
pMO196	pRS313	P_{ADH} *10MYC-SLI15(422-511 rk)*	This study
pMO205	pGEX-4T-1	GST-Bud2(1001-1104 T1092A T1101A)	This study
Rtt109	pET28a	6xHis-Rtt109	Kõivomägi et al., 2011
Sic1ΔC	pET28a	6xHis-Sic1(1-215)	Kõivomägi et al., 2011
Bop3	pET28a	6xHis-Bop3	Kõivomägi et al., 2011
Whi7	pET28a	6xHis-Whi7	Kõivomägi et al., 2013
Mms4	pET28a	6xHis-Mms4	This study
Hcm1	pET28a	6xHis-Hcm1	Kõivomägi et al., 2013
Gic2	pET28a	6xHis-Gic2	This study

Stb1	pET28a	6xHis-Stb1	Kõivomägi et al., 2011
Ash1	pET28a	6xHis-Ash1	Kõivomägi et al., 2011
Nrm1	pGEX-4T-1	GST-Nrm1	Kõivomägi et al., 2013
